# Increased Endoplasmic Reticulum Stress Response Is Involved in Clopidogrel-Induced Apoptosis of Gastric Epithelial Cells

**DOI:** 10.1371/journal.pone.0074381

**Published:** 2013-09-13

**Authors:** Hai-Lu Wu, Zhao-Tao Duan, Zong-Dan Jiang, Wei-Jun Cao, Zhi-Bing Wang, Ke-Wei Hu, Xin Gao, Shu-Kui Wang, Bang-Shun He, Zhen-Yu Zhang, Hong-Guang Xie

**Affiliations:** 1 Division of Gastroenterology, Department of Medicine, Nanjing First Hospital, Nanjing Medical University, Nanjing, China; 2 Central Laboratory, General Clinical Research Center, Nanjing First Hospital, Nanjing Medical University, Nanjing, China; 3 Division of Clinical Pharmacology, General Clinical Research Center, Nanjing First Hospital, Nanjing Medical University, Nanjing, China; 4 Department of Pharmacology, Nanjing Medical University School of Pharmacy, Nanjing, China; Universidade de Sao Paulo, Brazil

## Abstract

**Background:**

The widespread use of clopidogrel alone or in combination with aspirin may result in gastrointestinal mucosal injury, clinically represented as recurrent ulceration and bleeding complications. Our recent work suggested that clopidogrel significantly induced human gastric epithelial cell (GES-1) apoptosis and disrupted gastric mucosal barrier, and that a p38 MAPK inhibitor could attenuate such injury. However, their exact mechanisms are largely unknown.

**Methods:**

The GES-1 cells were used as a model system, the effects of clopidogrel on the whole gene expression profile were evaluated by human gene expression microarray and gene ontology analysis, changes of the mRNA and protein expression were determined by real-time PCR and Western blot analysis, and cell viability and apoptosis were measured by MTT assay and flow cytometry analysis, respectively.

**Results:**

Gene microarray analysis identified 79 genes that were differentially expressed (*P*<0.05 and fold-change >3) when cells were treated with or without clopidogrel. Gene ontology analysis revealed that response to stress and cell apoptosis dysfunction were ranked in the top 10 cellular events being affected, and that the major components of endoplasmic reticulum stress-mediated apoptosis pathway – CHOP and TRIB3– were up-regulated in a concentration- and time-dependent manner when cells were treated with clopidogrel. Pathway analysis demonstrated that multiple MAPK kinases were phosphorylated in clopidogrel-treated GES-1 cells, but that only SB-203580 (a p38-specific MAPK inhibitor) attenuated cell apoptosis and CHOP over-expression, both of which were induced by clopidogrel.

**Conclusions:**

Increased endoplasmic reticulum stress response is involved in clopidogrel-induced gastric mucosal injury, acting through p38 MAPK activation.

## Introduction

Clopidogrel, an antiplatelet agent, has been widely used to reduce the risk of cardiovascular events in patients with acute coronary syndromes (ACS) or those who underwent percutaneous coronary intervention (PCI) [1]. Concomitant use of clopidogrel and aspirin is a standardized dual antiplatelet therapy regimen for these patients. However, accumulated evidence has documented that the widespread use of clopidogrel is associated with a series of gastrointestinal (GI) side effects, such as recurrent gastric ulcer and GI bleeding complications [2]–[7]; however, the mechanism underlying clopidogrel-associated gastric mucosal injury has not been fully delineated.

Under normal circumstances, a dynamic balance between cell proliferation and apoptosis will maintain the integrity of gastric mucosal barrier. Studies have shown that apoptosis of gastric epithelial cells, induced by drugs, alcohol, *H. pylori* infection, and stress, is involved in the initiation and development of gastric mucosal injury [8]–[11], and that decreased apoptosis may result in attenuated gastric mucosal injury [12]–[14]. Therefore, further elucidation of the mechanism underlying clopidogrel-induced apoptosis would be helpful to better understand how clopidogrel could induce gastric mucosal lesions.

In human body, apoptosis may occur in response to various factors that exist simultaneously, rather than separately as they are investigated. In order to systematically identify which factors could be responsible for cell apoptosis, gene microarray analysis of the cultured cell is an optimal approach because many potential confounding factors could be minimized or even avoided *in vitro.* When cells are exposed to various stresses, including drugs, they would induce expression of a large number of proteins (so-called stress proteins) to protect themselves against stress-associated injury. When these stress proteins are severely impaired due to over-whelmed challenges, the cell organelle would elicit apoptotic signals, which may be associated with a variety of common diseases [15]–[18]. For example, in gastric epithelial cells, certain non-steroid anti-inflammatory drugs have been well demonstrated to induce endoplasmic reticulum (ER) stress response [19], resulting in increased cytosolic free Ca^2+^ levels [20] and/or increased oxidant stress [20], [21]. The ER is a cell organelle, where secretary proteins and membrane proteins are synthesized and folded. Correctly folded proteins in the ER are transported to the Golgi, whereas proteins that fail to be folded properly will be retained in the ER, and their further accumulation may constitute a form of stress to the affected cells (so-called “ER stress”) [22].

C/EBP homologous transcription factor (or called C/EBP homologous protein, also known as CHOP), or named as either DDIT3 (DNA damage induced transcript 3) or GADD153 (G1 arrest and DNA damage 153), is known to be involved in ER stress-induced apoptosis [23]. When the cell is experiencing ER stress, CHOP is significantly activated [24], [25] and is bound to C/EBP or Jun/Fos protein family to form a heterodimer, triggering apoptosis through regulating expression of apoptosis-related genes [26]. Up to date, a number of studies have demonstrated that the ER stress may lead to apoptosis or cell death through activation of MAPK (mitogen-activated protein kinase) family members, such as ERK (extracellular signal-regulated kinase), JNK (c-Jun- N-terminal kinase), and p38 [27], [28] in apoptotic pancreatic beta-cells, breast cancer cells, and gastric cancer cells [29]–[31].

In a recent report, we demonstrated that clopidogrel significantly induces apoptosis of human gastric epithelial cells (GES-1), disrupts cellular tight junction structure, and increases gastric epithelial permeability that could be partially abolished by the pretreatment of a p38 MAPK inhibitor [32]. However, the exact mechanism by which clopidogrel could induce apoptosis of gastric epithelial cells is largely unknown. In order to further delineate how clopidogrel could induce GES-1 cell apoptosis, we used an extensively recognized Agilent one-color microarray-based gene expression technique to measure altered mRNA expression in clopidogrel-treated gastric epithelial cells as compared with vehicle-treated cells and confirmed several most important genes involved.

## Materials and Methods

### Chemicals and Solutions

Clopidogrel powder (purity 99.18%), purchased from Beijing Nordhuns Chemical Technology Co. Ltd., China (lot # NDS11003), was dissolved in DMSO, whose final concentration present in working culture medium was restricted to be less than 0.1% (v/v) as used elsewhere [32]. Three MAPK-specific inhibitors – SB-203580, SP-600125, and U-0126– were purchased from Sigma (St Louis, MO, USA), and their working solutions were prepared as 1 µM in the culture medium containing less than 0.1% of DMSO. In addition, the working culture medium containing 0.1% DMSO was used as the vehicle control for all cell studies.

### The Cell Line Used in the Study

Human gastric epithelial cell line (also known as GES-1) with a phenotype similar to the gastric mucosal cell was obtained from the Shanghai Cell Bank, Chinese Academy of Sciences (Shanghai, China) [33]. DMEM-HG (Hyclone, Logan, UT, USA) was supplemented with 10% FBS and 1% antibiotics. The culture medium was changed every 48–72 h.

### MTT Assay

Cell proliferation or viability was determined with MTT (5 mg/ml, Sigma) as described elsewhere [32]. In brief, 20 µl MTT reagent was added into each well and incubated at 37°C for 4 h in the dark. The supernatant was aspirated, and formazan crystals were dissolved in 100 µl DMSO at 37°C for 10 min with gentle agitation. Absorbance of each sample was measured at 570 nm. Data were analyzed based on three independent experiments, and then normalized to the absorbance of the well that contained either media only (0%) or untreated cells (100%).

### Annexin V/Propidium Iodide Double Staining

Annexin V/propidium iodide double staining was used to detect apoptosis. GES-1 cells were plated in 60-mm dishes (3 ml, 1×10^6/^well) and incubated for 24 h at 37°C. After 24-h treatment with clopidogrel or an inhibitor of the MAPK, the cells were collected and washed twice with ice-cold PBS, and then were re-suspended in binding buffer at a concentration of 1×10^6^ cells/ml and incubated with 10 µl of PI (50 µg/ml) solution and 5 µl of FITC-conjugated AV (17.6 µg/ml) at 37°C for 5 min in the dark to achieve double staining. After staining, 400 µl of binding buffer were added to the cells, and then analyzed by flow cytometry (BD, FACSCanto™, USA).

### RNA Extraction and Purification

Total RNA was extracted from cultured cells using the single-step Trizol RNA extraction kit (Invitrogen, CA, USA), and its concentration and quality were determined by spectrophotometry and Agilent 2100 Bioanalyzer (Agilent Technologies, CA, USA) according to their respective manufacturer’s instructions. Only the samples that had no degradation were used to generate the labeled targets. Finally, total RNA was purified using an RNeasy mini kit (Qiagen).

### Microarray Hybridization

The Agilent Whole Human Genome Oligo Microarray (4×44 K, Agilent, San Diego, CA, USA), which represents more than 41, 000 human genes and transcripts, was used in this study to further systematically screen the differentially expressed genes between vehicle- and clopidogrel-treated cells. Single- and double-stranded cDNA was synthesized from total RNA samples (2 µg) according to Agilent Gene-Chip Expression Analysis Technical Manual. The cRNA was purified and fluorochrome labeled with Cy3, and then fragmented and hybridized to the gene chip at 65°C with rotation for 17 h. The Gene-Chips were washed and then scanned by Agilent scanner (G265BA; Agilent). Microarrays were provided by Shanghai Biochip Co. Ltd., China. All microarray datasets were submitted to the “Gene Expression Omnibus” with an accession number of GSE47591.

### Analysis of Differentially Expressed Genes

To elucidate the mechanisms of clopidogrel-induced apoptosis in gastric epithelial cells, the differentially expressed genes obtained from the primary analysis (see the Microarrays section) were further analyzed by SBC analysis system (http://www.ebioservice.com/), the web-based statistical software, provided by the Shanghai Biochip Co., Ltd. The core arithmetic of the SAS system was R software, which could accomplish the statistical analysis of the microarray data, combining seven public databases as summarized in **Table 1** to explore their biological meanings [34]. The significantly differentially expressed genes between vehicle-treated cells and clopidogrel-treated cells were identified based on the pre-specified criteria of a *P* value <0.05 and fold-change >3. The *P* value and FDR (false discovery rate) were calculated using the *t*-test modified from random variance model (RVM-*t*-test). FDR was calculated to correct each *P* value. The unsupervised hierarchical cluster analysis was performed using the Cluster 3.0 software (Berkeley, CA, USA).

**Table 1:** The websites of seven public databases used in this work.

Name of the web page	URL
NCBI Entrez Gene	http://www.ncbi.nlm.nih.gov/gene/
Gene Ontology	http://www.geneontology.org/
KEGG	http://www.genome.jp/kegg/
Biocarta	http://www.biocarta.com/
Human Protein Reference Database	http://www.hprd.org/
Molecular INTeraction database	http://mint.bio.uniroma2.it/mint/
Sanger microRNA	http://www.mirbase.org/

NCBI, National Center for Biotechnology Information; KEGG, Kyoto Encyclopedia of Genes and Genomes.

### Gene Ontology (GO) and Pathway Analysis

GO analysis, a key functional classification of NCBI, was used to analyze the main function of the differentially expressed genes. Pathway analysis was performed with the KEGG database. Two-side Fisher’s exact test and χ^2^ test were used to classify the GO category and pathway analysis; the FDR was calculated to correct each *P* value. A two-sided *P* value <0.05 was pre-specified as the threshold to determine statistically significant GO categories and KEGG pathways.

### Real-Time PCR Analysis of Target Genes

Total RNA was extracted using Trizol reagent (Invitrogen, CA, USA), and reverse transcription was carried out with M-MuLV reverse transcriptase (Fermentas) according to the manufacturer’s protocol, respectively. For PCR amplification, the primer sequences for CHOP and TRIB3 (the target gene each) as well as β-actin (an internal control gene) are given in **Table 2**. Real-time PCR was done using the ABI PRISM® 7500 Sequence Detection System (Applied Biosystems, Foster City, CA, USA) according to the manufacturer’s instruction. Finally, the comparative C_T_ (2^−ΔΔCT^) method was used to determine the relative concentration of the amplified products according to the instructions supplied by Applied Biosystems.

**Table 2:** Primer sequences for real-time PCR.

Gene	Primer sequence (5′→ 3′)
CHOP/DDIT3	F: gcc aaa atc aga gct gga acc t
	R: aca gtg tcc cga agg aga aag g
TRIB3	F: att agg cag ggt ctg tcc tgt g
	R: agt atg gac ctg gga ttg tgg a
β-actin	F: gcg gga aat cgt gcg tga cat t
	R: cta cct caa ctt cca tca aag cac

F, forward; R, reverse.

### Western Blot Analysis

Western blot analysis was performed following the procedure as described elsewhere [32]. Briefly, cell lysates with equal amount of proteins were loaded, separated by SDS-PAGE gels, and transferred onto the nitrocellulose membrane. That membrane was incubated with specific primary antibodies (1∶1500) (CST, Beverley, MA, USA) at 4°C overnight, followed by appropriate horseradish peroxidase-conjugated secondary antibodies (CST, Beverley, MA, USA) at ambient temperature for 2 h. Protein expression was semi-quantified by Image J software (NIH, MD, USA). All experiments were done three times.

### Statistical Analysis

For MTT assay, real-time PCR, and Western blot analysis, statistical analysis was performed by use of SPSS 13.0 (Chicago, IL, USA). All data are expressed as mean ± SD. Data were analyzed using one-way ANOVA, followed by either the LSD procedure (if variance was equal) or the Games-Howell procedure (if variance was unequal). A two-sided *P* value <0.05 was considered statistically significant.

## Results

### Differentially Expressed Genes in Vehicle- and Clopidogrel-Treated Cells

**Figure 1:**
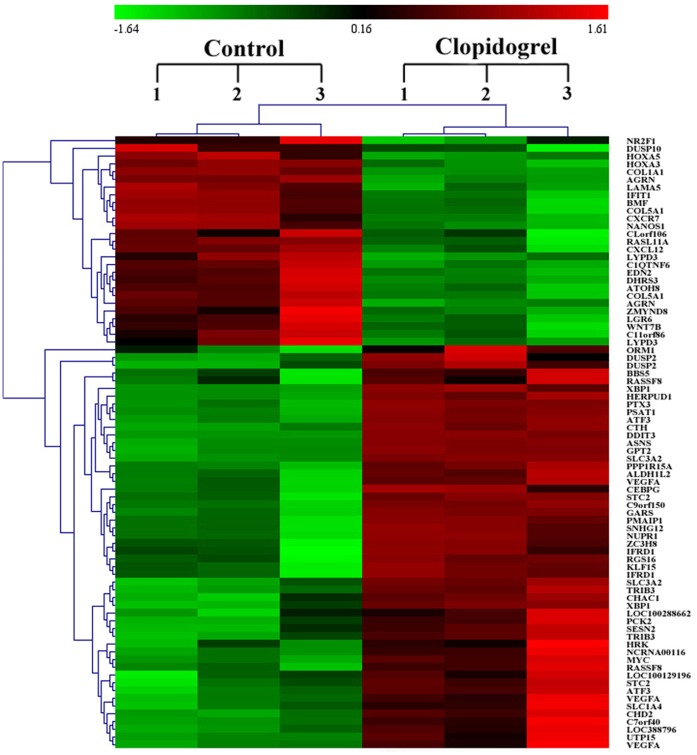
Hierarchical cluster analysis of 79 differentially expressed genes in all 6 samples. Hierarchical cluster analysis was performed as described in the Materials and Methods section. Each column represents one sample, and each gene is depicted by one row, where red denotes an increase in gene expression and green denotes a decrease in gene expression as compared with the other group. The brighter the color, the higher the gene expression level.

To gain insights into the mechanisms underlying the pro-apoptotic effect of clopidogrel, GES-1 cells were cultivated in the absence or presence of 1.5 mM clopidogrel for 24 h, followed by the Agilent Whole Human Genome Oligo Microarray. A total of 79 genes were found to be differentially expressed between vehicle- and clopidogrel-treated GES-1 cells (*P*<0.05, and fold-change >3). A heat map with two-dimensional hierarchical clustering revealed 79 genes differentially expressed between the two groups as illustrated in **Figure 1**. Of them, 52 genes were up-regulated, and 27 were down-regulated in clopidogrel-treated cells as compared with the vehicle-treated control cells.

### GO and Pathway Analysis of Differentially Expressed Genes

**Figure 2:**
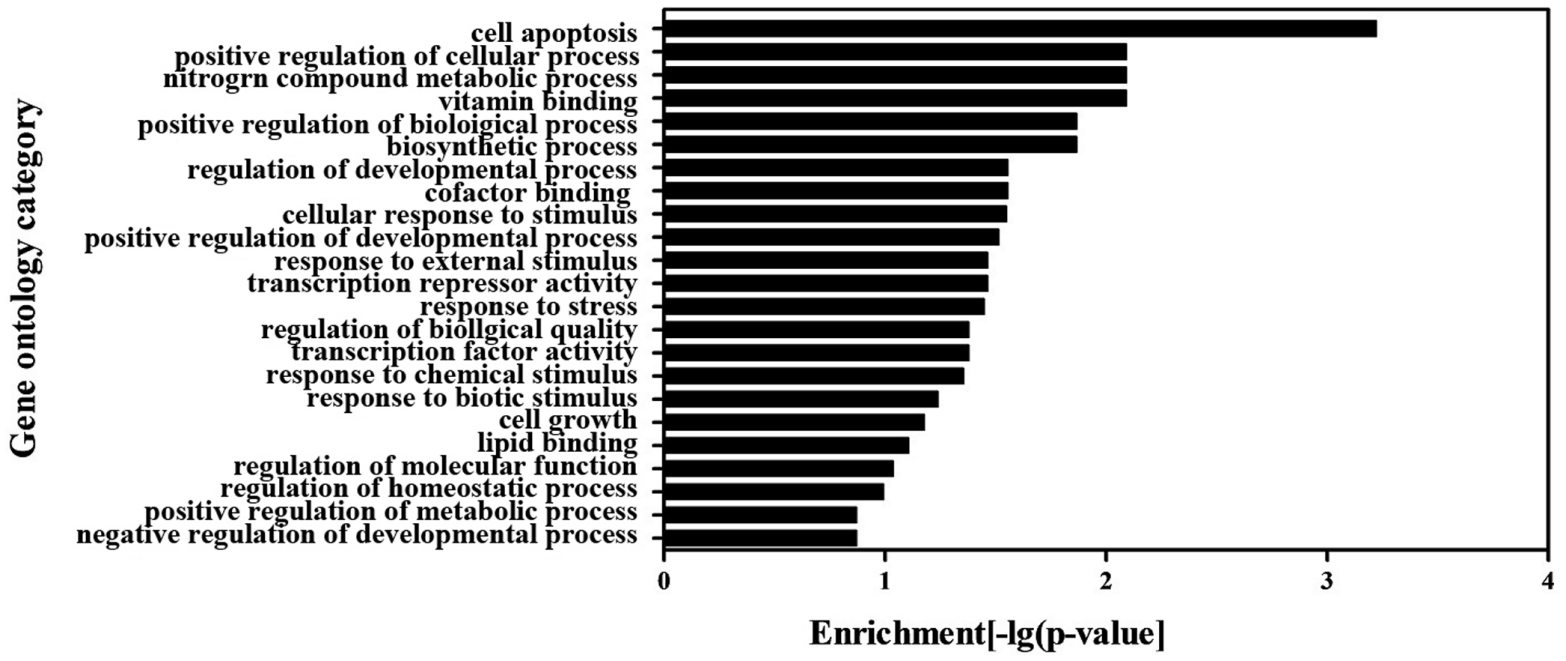
The GO category for the up-regulated genes in clopidogrel group. A *P* value <0.05 was used as a cut-off threshold to select significant GO categories. The higher the enrichment, the more significant the biological processes.

**Figure 3:**
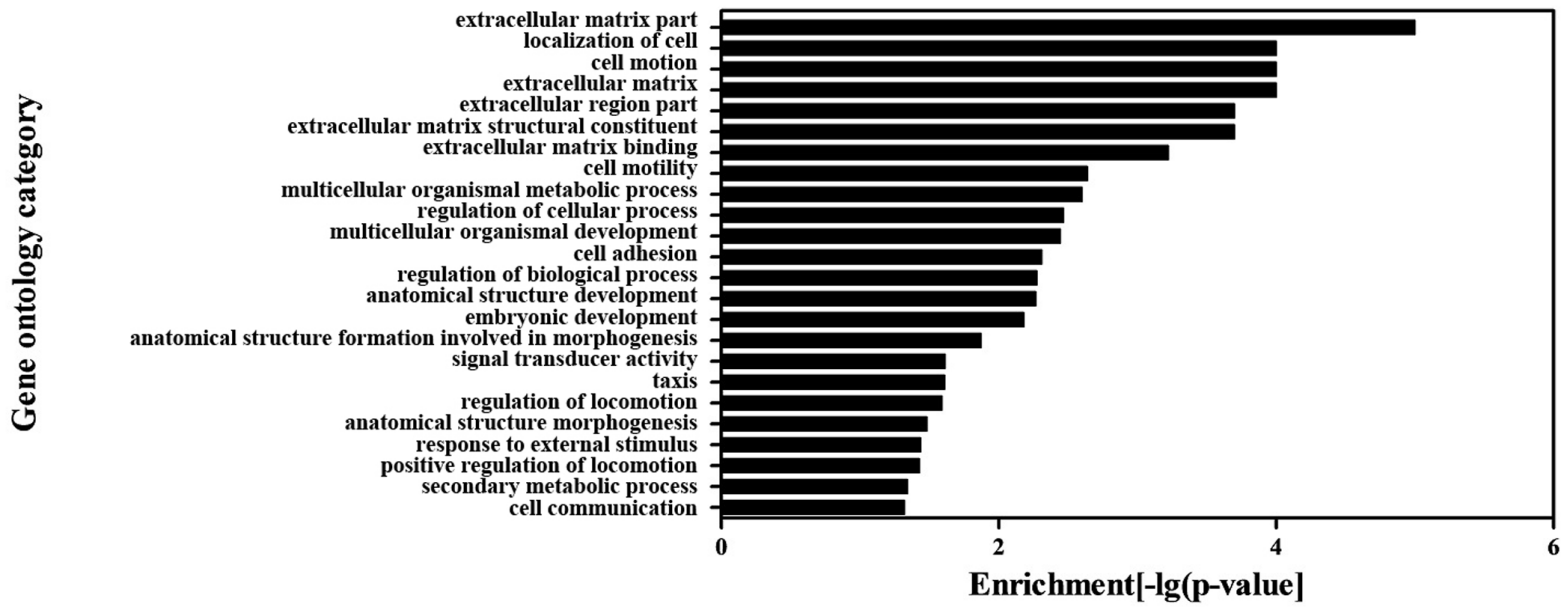
The GO category for the down-regulated genes in clopidogrel group. A *P* value <0.05 was used as a cut-off threshold to select significant GO categories. The higher the enrichment, the more significant the biological processes.

To further elucidate the potential mechanisms by which clopidogrel could induce apoptosis of gastric epithelial cells, a group of differentially expressed genes obtained from the primary analysis (see above) were analyzed by GO enrichment and pathway analysis, respectively. The GO enrichment analysis revealed that the main GO categories for the up-regulated genes in clopidogrel-treated cells included cell apoptosis, cell growth, cellular response to stimulus (such as external stimulus, or stress), metabolic process of nitrogen compound, positive regulation of cellular process and so on as summarized in **Figure 2**. Among the down-regulated genes, extracellular matrix part, cell motion, multicellular organismal metabolic process, regulation of biological process, signal transducer activity, response to external stimulus and others were enriched as shown in **Figure 3**. Subsequently, the KEGG database was used to investigate the pathways where these differentially expressed genes are located at. The significantly affected target pathways were designated as those with a *P* value <0.05. A total of 17 pathways were identified, and top 10 related genes are summarized in **Table 3**. Among them, apoptosis-related pathways – MAPKs – were activated significantly, leading to up-regulation of stress responsive transcriptional regulator CHOP.

**Table 3:** The top 10 pathways that could be affected by clopidogrel in the GES-1 cells.

Pathway	*P*-value*	FDR**	Gene involved
1. ECM-receptor interaction	0	0	AGRN, COL1A1, COL5A1, LAMA5
2. Focal adhesion	2.00E-04	1.00E-04	COL1A1, COL5A1, LAMA5, **VEGFA**
3. MAPK signaling pathway	6.00E-04	2.00E-04	**DDIT3**, DUSP10, **DUSP2**, **MYC**
4. Nitrogen metabolism	6.00E-04	2.00E-04	**ASNS**, **CTH**
5. Alanine, aspartate and glutamatemetabolism	0.001	2.00E-04	**ASNS**, **GPT2**
6. Glycine, serine and threonine metabolis	0.001	2.00E-04	**CTH**, **PSAT1**
7. Pathways in cancer	0.0012	2.00E-04	LAMA5, **MYC**, **VEGFA**, WNT7B
8. Bladder cancer	0.0017	3.00E-04	**MYC**, **VEGFA**
9. p53 signaling pathway	0.0044	5.00E-04	**PMAIP1**, **SESN2**
10. Metabolic pathways	0.0046	5.00E-04	**ASNS**, **CTH**, DHRS3, **GPT2**, **PCK2**

* Enrichment *P*-value of the corresponding pathway as determined by Fisher’s exact test.

** FDR of the corresponding pathway.

Bold, up-regulated; non-bold, down-regulated.

In this experiment, the genes responsible for cell apoptosis, growth, and response to stress were most important to match our research goal. Analysis of our microarray data showed that 13 genes associated with stress response (shown in **Table 4**) and 16 genes associated with cell apoptosis and growth (**Table 5**) were markedly altered after GES-1 cells were treated with clopidogrel.

**Table 4:** Microarray analysis for the differentially expressed genes involved in ER stress.

Gene ID	Gene symbol	Name of the gene (or gene product)	Fold-change*
A_23_P21134	**CHOP/DDIT3**	DNA-damage-inducible transcript 3	21.79
A_23_P210690	**TRIB3**	tribbles homolog 3 (Drosophila)	14.97
A_23_P145694	ASNS	asparagine synthetase	7.27
A_23_P121064	PTX3	pentraxin-related gene, rapidly induced by IL-1 beta	5.08
A_23_P207520	COL1A1	collagen, type I, alpha 1	−4.55
A_23_P25194	HRK	harakiri, BCL2 interacting protein (contains only BH3 domain)	4.48
A_23_P356755	CEBPG	CCAAT/enhancer binding protein (C/EBP), gamma	4.47
A_23_P70398	VEGFA	vascular endothelial growth factor A	4.36
A_23_P90172	PPP1R15A	protein phosphatase 1, regulatory (inhibitor) subunit 15A	3.7
A_23_P158593	COL5A1	collagen, type V, alpha 1	−3.7
A_23_P169494	ORM1	orosomucoid 1	3.57
A_23_P54846	HERPUD1	homocysteine-inducible, endoplasmic reticulum stress-inducible,ubiquitin-like domain member 1	3.33
A_24_P182494	DUSP10	dual specificity phosphatase 10	−3.13

* Fold-change, clopidogrel/control; minus sign (−) denotes down-regulated genes.

**Table 5:** Microarray analysis for the differentially expressed genes involved in cell apoptosis and growth.

Gene ID	Gene symbol	Gene description	Fold-change*
**Apoptosis-related genes**			
A_23_P21134	**CHOP/DDIT3**	DNA-damage-inducible transcript 3	21.79
A_23_P210690	**TRIB3**	tribbles homolog 3 (Drosophila)	14.97
A_24_P270728	NUPR1	nuclear protein 1	10.92
A_23_P145694	ASNS	asparagine synthetase	7.27
A_23_P25194	HRK	harakiri, BCL2 interacting protein (contains only BH3 domain)	4.48
A_23_P356755	CEBPG	CCAAT/enhancer binding protein (C/EBP), gamma	4.47
A_23_P70398	VEGFA	vascular endothelial growth factor A	4.36
A_23_P379649	BMF	Bcl2 modifying factor	−4.35
A_23_P90172	PPP1R15A	protein phosphatase 1, regulatory (inhibitor) subunit 15A	3.7
A_23_P108871	ZC3H8	zinc finger CCCH-type containing 8	3.43
A_24_P154948	GARS	glycyl-tRNA synthetase	3.39
A_23_P54846	HERPUD1	homocysteine-inducible, endoplasmic reticulum stress-inducible,ubiquitin-like domain member 1	3.33
A_23_P207999	PMAIP1	phorbol-12-myristate-13-acetate-induced protein 1	3.22
**Cell growth-related genes**			
A_24_P270728	NUPR1	nuclear protein 1	10.92
A_23_P126103	CTH	cystathionase (cystathionine gamma-lyase)	7.66
A_23_P75811	SLC3A2	solute carrier family 3 (activators of dibasic and neutralamino acid transport), member 2	4.35

* Fold-change, clopidogrel/control; minus sign (−) denotes a down-regulated gene.

### Clopidogrel-Induced the ER Stress in GES-1 Cells

**Figure 4:**
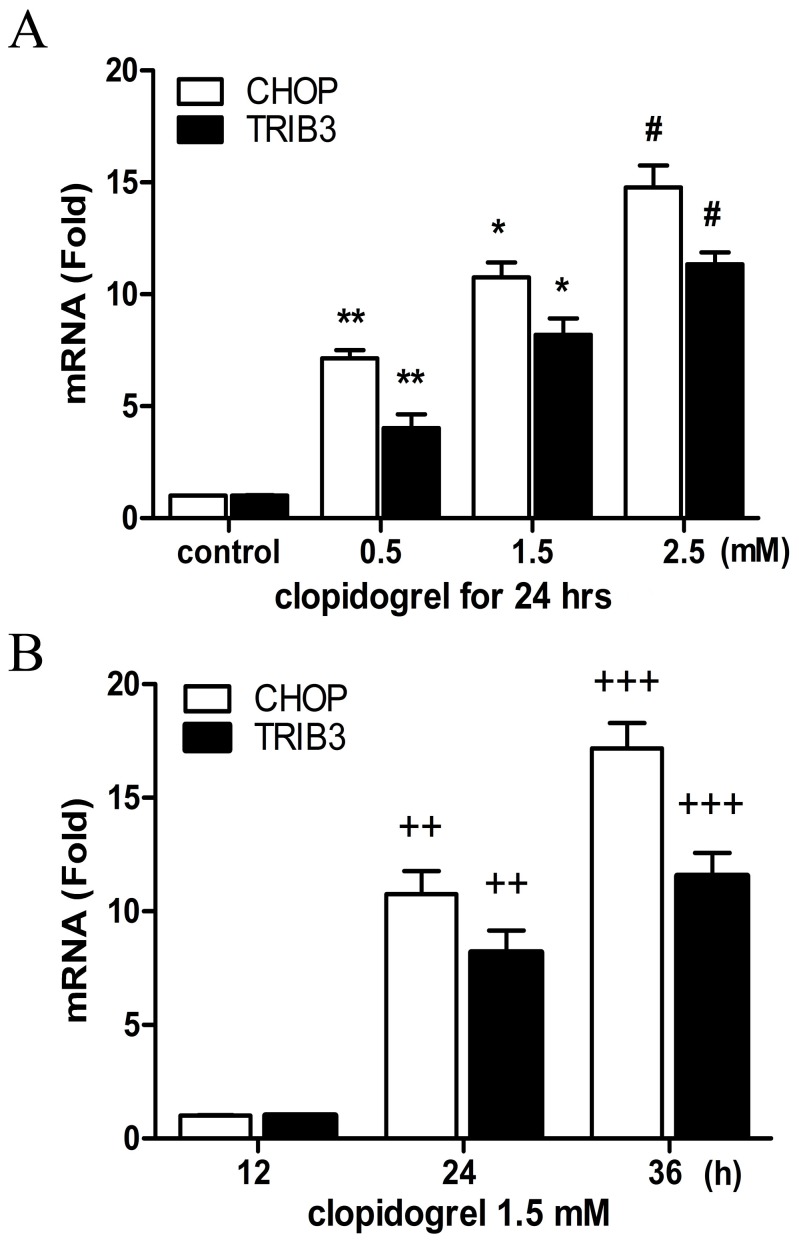
Effects of clopidogrel on CHOP and TRIB3 mRNA expression in the GES-1 cells. The mRNA expression levels of both CHOP and TRIB3 were up-regulated in the GES-1 cells in a concentration- and time-dependent manner when treated with clopidogrel, as measured by real-time PCR. Data are a representative of three independent experiments. **P<0.05 *vs* vehicle control; *P<0.05 *vs* clopidogrel (0.5 mM); #*P<*0.05 *vs* clopidogrel (1.5 mM); *^++^P<*0.05 *vs* clopidogrel (1.5 mM for 12 h); *^+++^P<*0.05 *vs* clopidogrel (1.5 mM for 24 h).

**Figure 5:**
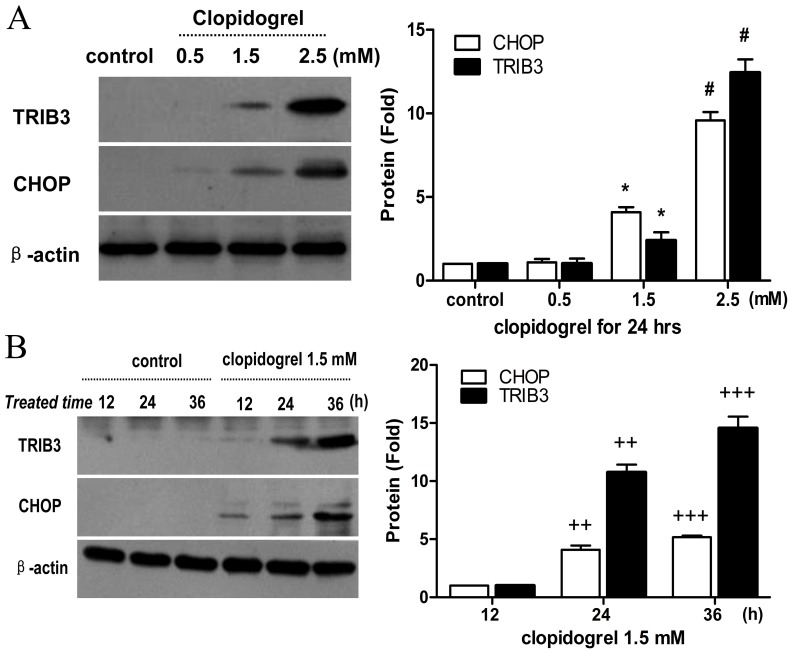
Effects of clopidogrel on CHOP and TRIB3 protein expression in the GES-1 cells. The protein expression levels of both CHOP and TRIB3 were up-regulated in the GES-1 cells in a concentration- and time-dependent manner, consistent with their mRNA expression profiles as measured by real-time PCR. Data are a representative of three independent experiments. **P<0.05 *vs* vehicle control; #*P<*0.05 *vs* clopidogrel (1.5 mM); *^++^P<*0.05 *vs* clopidogrel (1.5 mM for 12 h); *^+++^P<*0.05 *vs* clopidogrel (1.5 mM for 24 h).

In this study, cell apoptosis was found to be enriched for the genes that encode several stress responsive transcriptional regulators like CHOP and TRIB3, further pointing to the role of the ER stress in clopidogrel-induced apoptosis. The most significant ER stress-induced apoptotic pathway is mediated through CHOP. The microarray data showed a 21.79-fold increase in CHOP and a 14.97-fold increase in TRIB3 levels after GES-1 cells were treated with clopidogrel for 24 h as shown in **Table 4**. TRIB3, a novel target of CHOP, is known to be involved in CHOP-dependent cell death during the ER stress [35]. In this study, we observed both TRIB3 and CHOP were up-regulated by clopidogrel in the GES-1 cells in a concentration- and time-dependent manner, as measured by real-time PCR and Western blot analysis (**Figures 4** and **5**), consistent with our previous observations [32].

**Figure 6:**
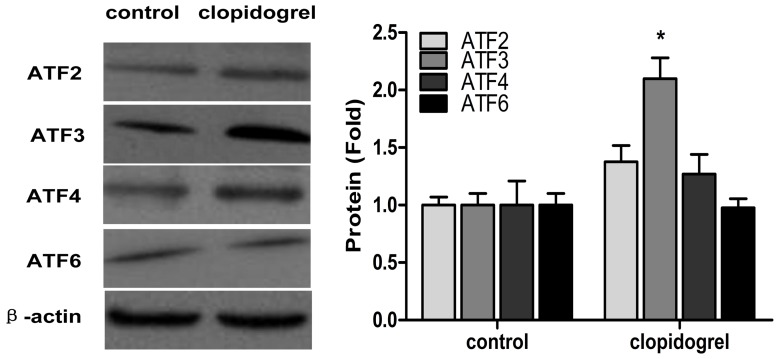
Effects of ER stress on ATF expression in the GES-1 cells. GES-1 cells were treated with clopidogrel 1.5 mM for 24 h. Data are expressed as mean ± SD, representative of three independent experiments. *P<0.05 *vs* control.

Since the expression of CHOP is regulated at the transcriptional level through the upstream transcription factor ATFs, we also determined expression of ATF2, ATF3, ATF4, and ATF6 at protein levels in the GES-1 cells. As expected, there was >2-fold increased protein expression of ATF3 in clopidogrel-treated GES-1 cells as compared with controls. In contrast, there were no changes in mRNA and protein expression of ATF2, ATF4, and ATF6, respectively (**Figure 6**).

### The p38 MAPK Inhibitor SB-203580 Attenuated Clopidogrel-Induced GES-1 Cell Apoptosis and CHOP Up-regulation

**Figure 7:**
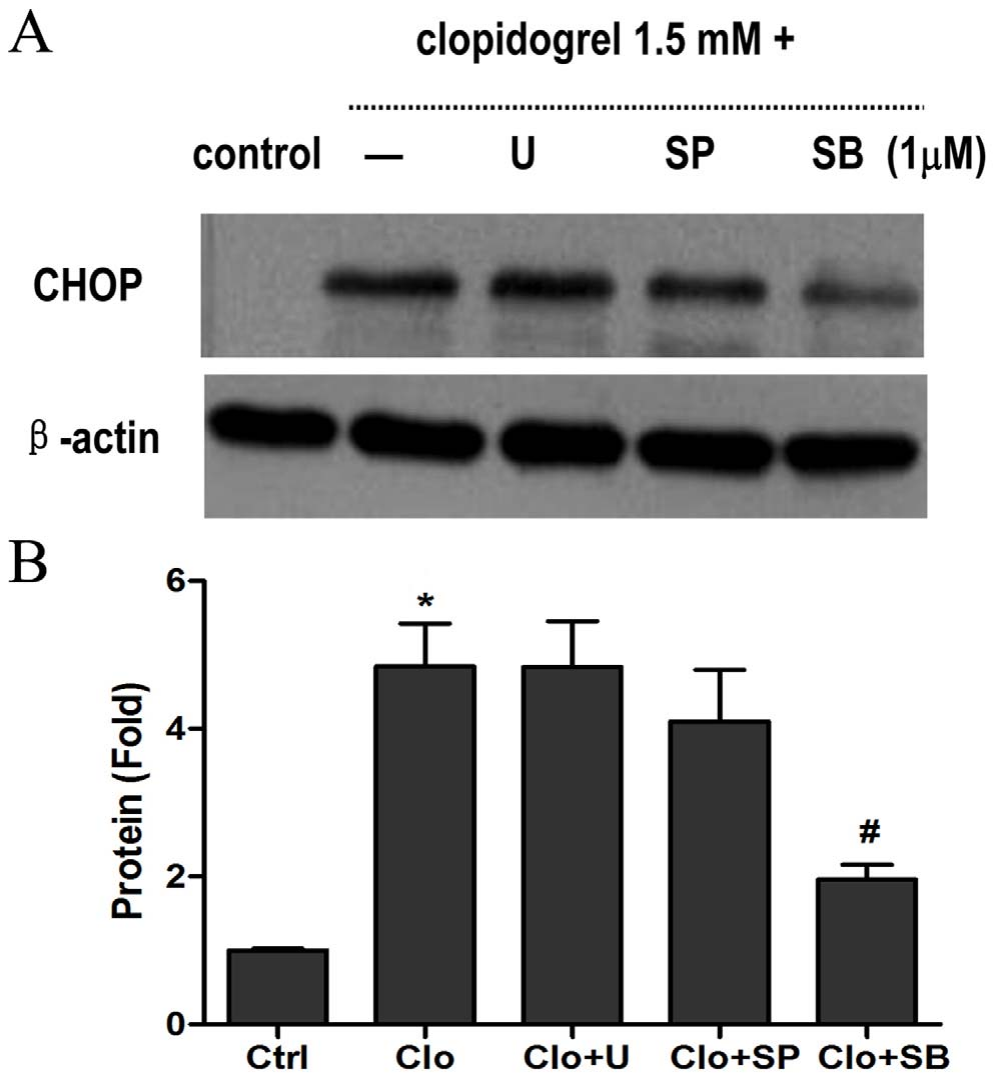
Suppression of clopidogrel-induced CHOP up-regulation by the p38 MAPK inhibitor. As expected, only the p38 MAPK inhibitor SB-203580 significantly attenuated CHOP up-regulation. Data are expressed as mean ± SD, representative of three independent experiments. *P<0.05 *vs* control; #*P<*0.05 *vs* clopidogrel alone (1.5 mM).

**Figure 8:**
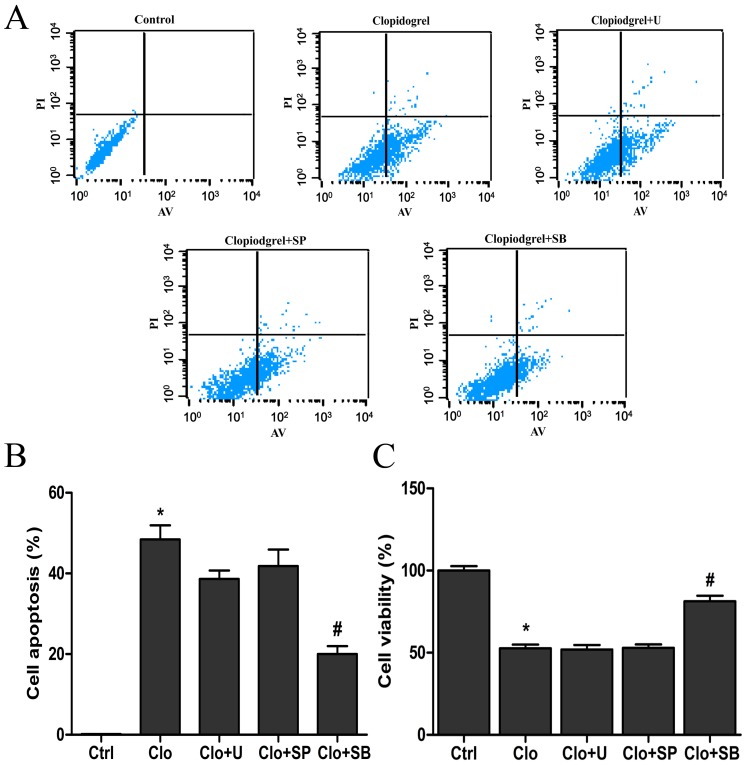
The p38 MAPK inhibitor suppressed clopidogrel-induced cell proliferation inhibition and apoptosis. After pretreatment of a p38 MAPK inhibitor SB-203580, clopidogrel-induced cell proliferation inhibition and apoptosis were significantly attenuated. Data are expressed as mean ± SD, representative of three independent experiments. *P<0.05 *vs* control; #*P*<0.05 *vs* clopidogrel alone.

To investigate whether MAPK activation could contribute to ER stress-induced GES-1 cell apoptosis after exposure to clopidogrel, three MAPK-specific inhibitors were used. Pretreatment with the p38 MAPK inhibitor SB-203580 (1 µM) for 30 min significantly attenuated over-expression of CHOP (**Figure 7**), cell apoptosis and inhibition of cell proliferation, all of which were induced by clopidogrel (1.5 mM for 24 h), but pretreatment with either the ERK inhibitor U-0126 (1 µM) or the JNK inhibitor SP-600125 (1 µM) had no marked effects (**Figure 8**). In contrast, there were no marked changes in cell viability and apoptosis, and CHOP expression in GES-1 cells when pretreated with SB-203508, U-0126 or SP-600125 (1 µM for 24.5 h, respectively) in the absence of clopidogrel treatment (data not shown).

## Discussion

To better understand cellular responses to clopidogrel and potential signaling pathways activated by clopidogrel, the differentially expressed genes were determined in the absence or presence of clopidogrel treatment by the Agilent one-color microarray-based gene expression profiling. The major findings in this study were that some genes associated with ER stress (such as ATF3, CHOP, and TRIB3) are over-expressed in the GES-1 cells when treated with clopidogrel, and that the p38/MAPK inhibitor SB-203580 can partially abolish GES-1 apoptosis and CHOP over-expression, both of which are induced by clopidogrel.

CHOP is a key regulator of the ER-stress response [23]. Under normal conditions, the level of CHOP in the cytoplasm is very low [36]. When the ER stress response is triggered by certain cellular stress, such as hypoxia, oxidant stress, glucose/nutrient starvation, and drugs, CHOP would be induced and transferred from cytoplasm to nucleus to regulate expression of its target genes that may potentiate apoptosis [37]–[39]. These target genes include BIM (BCL2-like 11) [40], ERO1-Lα (endoplasmic oxidoreductin-1-like) [40], GADD34 (growth arrest and DNA damage gene 34) [41], and TRIB3 [35]. Tsutsumi et al observed that indomethacin-induced apoptosis was suppressed in cultured guinea-pig gastric mucosal cells by expression of the dominant-negative form of CHOP, or in peritoneal macrophages from CHOP-deficient mice [19]. To analyze the ER stress response in clopidogrel-induced GES-1 cell apoptosis, we performed real-time PCR and Western blot analysis, and found that expression of CHOP and its target gene TRIB3 was up-regulated in the GES-1 cells in a concentration- and time-dependent manner in response to clopidogrel (**Figures 4** and **5**), consistent with the results of cell viability and apoptosis experiments in our previous study [32]. These data suggest that the induction of these genes/proteins may be important in apoptosis of gastric epithelial cell GES-1 induced by clopidogrel.

TRIB3, one of the CHOP target genes, can interact with CHOP, but does not promote degradation of CHOP protein [35]. Some ER-stress inducers, such as tunicamycin, cannabinoids, thapsigargin and nutrient starvation, can increase expression of both CHOP and TRIB3 [42], [43], leading to apoptosis. In this experiment, we found that the levels of CHOP increased with TRIB3 when apoptosis occurred, and that the peak of TRIB3 expression was later than that of CHOP expression as shown in **Figure 5**. TRIB3 is also known to cause apoptosis through inhibiting Akt kinase activity, an anti-apoptotic factor kinase by altering the phosphorylation of Thr308 and Ser473 [44]. In most cases, the expression of CHOP in response to stress is regulated at the transcriptional level through its upstream transcription factors ATF2, ATF4, and ATF6 [37], [42], [45]. As shown in **Figure 6**, clopidogrel did up-regulate ATF3, but not ATF2, ATF4, and ATF6, consistent with the findings of others [46]–[48]. Thus, which ATFs could regulate CHOP expression may vary by the type of the cell or tissue being studied or the stimulant used.

The first step to respond to ER stress is to synthesize a large amount of proteins that may contribute to protein folding through transcriptional machinery, because the unfolded proteins accumulated in the ER would be degraded easily. If such responsiveness failed, the intent would activate MAPKs and/or nuclear factor κB (NFκB) that induce expression of the genes that encode the mediators of host defense [49]. If adaptation and alarm all failed to get rid of ER stress, the cell would undergo apoptosis [50]. Mauro et al demonstrated that ticlopidine, the first-generation P2Y12 receptor antagonist, could induce endothelial cell apoptosis by disrupting production of extracellular matrix components critical to microvascular endothelial cell integrity in vitro, and that ticlopidine-induced apoptosis could be abrogated by inhibitors of ERK1/2 and p38 phosphorylation [51]. However, the mechanisms remain to be determined. In a recent study, we found that ERK, JNK, and p38 MAPKs all were markedly activated in clopidogrel-treated GES-1 cells as compared with the vehicle-treated controls, but that only the p38 MAPK inhibitor (SB203580) could attenuate damaged tight junction structure and increased paracellular permeability, which of both were induced by clopidogrel [32]. Furthermore, apoptosis of gastric mucosal epithelial cells has been demonstrated to be the important pathological basis for promoting the occurrence of gastric epithelial barrier dysfunction [52], [53]. Therefore, in this work, we sought to systematically screen how many genes would be responsible for clopidogrel-induced gastric epithelial cell apoptosis, and to further determine which genes would be the most important and whether the p38 MAPK could be also involved in them. This study demonstrated that p38 MAPK inhibitor SB203580 could suppress GES-1 apoptosis and CHOP over-expression (as shown in **Figure 7**), both of which were induced by clopidogrel, consistent with previous findings [54]–[56]. The p38 MAPK, a highly conserved proline-directed serine/threonine protein kinase, plays an important role in mediating stress, inflammatory and immune response, cell survival and apoptosis processes [57], [58]. Moreover, it has been recognized that two adjacent serine residues (Ser^79^ and Ser^82^) of CHOP can serve as substrates of the p38 MAPK family [55], [59]. In this study, the p38 MAPK inhibitor suppressed GES-1 cell apoptosis and CHOP over-expression, indicating that p38 MAPK activation may play a critical role in CHOP-mediated gastric epithelial cell apoptosis induced by clopidogrel.

There are also studies demonstrating that, besides CHOP-mediated apoptotic pathways, IRE1-mediated activation of ASK1 (apoptosis signal-regulating kinase 1)/JNK and activation of caspase-12 are also the major mechanisms of ER stress-induced apoptosis [23]. In this experiment, we found that the JNK kinase inhibitor could not alleviate gastric epithelial cell apoptosis. Based on this evidence, it is concluded that JNK signaling pathway seems not to be involved in clopidogrel-induced gastric epithelial cell apoptosis. In addition, caspase-12, a marker of ER stress-induced apoptosis in mouse, can be activated by ER stress [60]. Caspase-12, in turn, activates caspase-9 and caspase-3, leading to cell death. Because humans lack functional caspase-12 homologue [60], caspase-12 was not measured in this study.

In summary, this study demonstrated that the up-regulation of ATF3, CHOP, and TRIB3 is the result of clopidogrel treatment in the GES-1 cells, whose increased expression can lead to ER stress and gastric epithelial cellular apoptosis through the activation of the p38 MAPK signaling pathway. In terms of the widespread use of clopidogrel in patient care, there is the need to further elucidate the mechanism underlying clopidogrel-induced GI complications.

## References

[1] Xie HG, Zou JJ, Hu ZY, Zhang JJ, Ye F, et al (2011) Individual variability in the disposition of and response to clopidogrel: Pharmacogenomics and beyond. Pharmacol Ther 129: 267–289. 20965214 10.1016/j.pharmthera.2010.10.001

[2] Chan FK, Ching JY, Hung LC, Wong VW, Leung VK, et al (2005) Clopidogrel versus aspirin and esomeprazole to prevent recurrent ulcer bleeding. N Engl J Med 352: 238–244. 15659723 10.1056/NEJMoa042087

[3] Taha AS, Angerson WJ, Knill-Jones RP, Blatchford O (2006) Upper gastrointestinal mucosal abnormalities and blood loss complicating low-dose aspirin and antithrombotic therapy. Aliment Pharmacol Ther 23: 489–495. 16441469 10.1111/j.1365-2036.2006.02784.x

[4] Ziegelin M, Hoschtitzky A, Dunning J, Hooper T (2007) Does clopidogrel rather than aspirin plus a proton-pump inhibitor reduce the frequency of gastrointestinal complications after cardiac surgery? Interact Cardiovasc Thorac Surg 6: 534–537. 17669930 10.1510/icvts.2007.157941

[5] Shmulevich E, Friger M, Gilutz H, Azab AN (2011) Clopidogrel and proton pump inhibitors: is there a significant drug-drug interaction? Can J Cardiovasc Nurs 21: 27–36. 22165503

[6] Shin JS, Abah U (2012) Is routine stress ulcer prophylaxis of benefit for patients undergoing cardiac surgery? Interact Cardiovasc Thorac Surg 14: 622–628. 22345061 10.1093/icvts/ivs019 PMC3735850

[7] Takayama S, Izuhara C, Yamada N, Yamanaka S, Hashimoto E, et al (2012) A new model of gastric bleeding induced in rats by aspirin plus clopidogrel under stimulation of acid secretion. Prophylactic effects of antiulcer drugs. J Physiol Pharmacol 63: 41–52. 22460460

[8] Choi SR, Lee SA, Kim YJ, Ok CY, Lee HJ, et al (2009) Role of heat shock proteins in gastric inflammation and ulcer healing. J Physiol Pharmacol 60 Suppl 75–17. 20388941

[9] Chiou SK, Hodges A, Hoa N (2010) Suppression of growth arrest and DNA damage-inducible 45alpha expression confers resistance to sulindac and indomethacin-induced gastric mucosal injury. J Pharmacol Exp Ther 334: 693–702. 20498252 10.1124/jpet.110.168153

[10] Gomi A, Harima-Mizusawa N, Shibahara-Sone H, Kano M, Miyazaki K, et al (2013) Effect of Bifidobacterium bifidum BF-1 on gastric protection and mucin production in an acute gastric injury rat model. J Dairy Sci 96: 832–837. 23200466 10.3168/jds.2012-5950

[11] Saberi S, Douraghi M, Azadmanesh K, Shokrgozar MA, Zeraati H, et al (2012) A potential association between Helicobacter pylori CagA EPIYA and multimerization motifs with cytokeratin 18 cleavage rate during early apoptosis. Helicobacter 17: 350–357. 22967118 10.1111/j.1523-5378.2012.00954.x

[12] Park S, Hahm KB, Oh TY, Jin JH, Choue R (2004) Preventive effect of the flavonoid, wogonin, against ethanol-induced gastric mucosal damage in rats. Dig Dis Sci 49: 384–394. 15139485 10.1023/b:ddas.0000020490.34220.6d

[13] Jainu M, Devi CS (2006) Gastroprotective action of Cissus quadrangularis extract against NSAID induced gastric ulcer: role of proinflammatory cytokines and oxidative damage. Chem Biol Interact 161: 262–270. 16797507 10.1016/j.cbi.2006.04.011

[14] Laine L, Takeuchi K, Tarnawski A (2008) Gastric mucosal defense and cytoprotection: bench to bedside. Gastroenterology 135: 41–60. 18549814 10.1053/j.gastro.2008.05.030

[15] Lou LX, Geng B, Yu F, Zhang J, Pan CS, et al (2006) Endoplasmic reticulum stress response is involved in the pathogenesis of stress induced gastric lesions in rats. Life Sci 79: 1856–1864. 16875701 10.1016/j.lfs.2006.06.022

[16] Meir O, Dvash E, Werman A, Rubinstein M (2010) C/EBP-beta regulates endoplasmic reticulum stress-triggered cell death in mouse and human models. PLoS One 5: e9516. 20209087 10.1371/journal.pone.0009516 PMC2831074

[17] Schroder M, Sutcliffe L (2010) Consequences of stress in the secretory pathway: The ER stress response and its role in the metabolic syndrome. Methods Mol Biol 648: 43–62. 20700704 10.1007/978-1-60761-756-3_3

[18] Raghubir R, Nakka VP, Mehta SL (2011) Endoplasmic reticulum stress in brain damage. Methods Enzymol 489: 259–275. 21266235 10.1016/B978-0-12-385116-1.00015-7

[19] Tsutsumi S, Gotoh T, Tomisato W, Mima S, Hoshino T, et al (2004) Endoplasmic reticulum stress response is involved in nonsteroidal anti-inflammatory drug-induced apoptosis. Cell Death Differ 11: 1009–1016. 15131590 10.1038/sj.cdd.4401436

[20] Lim MS, Lim PL, Gupta R, Boelsterli UA (2006) Critical role of free cytosolic calcium, but not uncoupling, in mitochondrial permeability transition and cell death induced by diclofenac oxidative metabolites in immortalized human hepatocytes. Toxicol Appl Pharmacol 217: 322–331. 17097122 10.1016/j.taap.2006.09.012

[21] Omatsu T, Naito Y, Handa O, Hayashi N, Mizushima K, et al (2009) Involvement of reactive oxygen species in indomethacin-induced apoptosis of small intestinal epithelial cells. J Gastroenterol 44 Suppl 1930–34. 19148790 10.1007/s00535-008-2293-3

[22] Zhang K, Kaufman RJ (2008) From endoplasmic-reticulum stress to the inflammatory response. Nature 454: 455–462. 18650916 10.1038/nature07203 PMC2727659

[23] Malhotra JD, Kaufman RJ (2007) Endoplasmic reticulum stress and oxidative stress: a vicious cycle or a double-edged sword? Antioxid Redox Signal 9: 2277–2293. 17979528 10.1089/ars.2007.1782

[24] Ubeda M, Habener JF (2000) CHOP gene expression in response to endoplasmic-reticular stress requires NFY interaction with different domains of a conserved DNA-binding element. Nucleic Acids Res 28: 4987–4997. 11121490 10.1093/nar/28.24.4987 PMC115245

[25] Tang JR, Nakamura M, Okura T, Takata Y, Watanabe S, et al (2002) Mechanism of oxidative stress-induced GADD153 gene expression in vascular smooth muscle cells. Biochem Biophys Res Commun 290: 1255–1259. 11811998 10.1006/bbrc.2002.6336

[26] Corazzari M, Lovat PE, Oliverio S, Di Sano F, Donnorso RP, et al (2005) Fenretinide: a p53-independent way to kill cancer cells. Biochem Biophys Res Commun 331: 810–815. 15865936 10.1016/j.bbrc.2005.03.184

[27] Ramirez-Alcantara V, LoGuidice A, Boelsterli UA (2009) Protection from diclofenac-induced small intestinal injury by the JNK inhibitor SP600125 in a mouse model of NSAID-associated enteropathy. Am J Physiol Gastrointest Liver Physiol 297: G990–998. 20501447 10.1152/ajpgi.00219.2009

[28] Choi CH, Jung YK, Oh SH (2010) Autophagy induction by capsaicin in malignant human breast cells is modulated by p38 and extracellular signal-regulated mitogen-activated protein kinases and retards cell death by suppressing endoplasmic reticulum stress-mediated apoptosis. Mol Pharmacol 78: 114–125. 20371669 10.1124/mol.110.063495

[29] Feng R, Zhai WL, Yang HY, Jin H, Zhang QX (2011) Induction of ER stress protects gastric cancer cells against apoptosis induced by cisplatin and doxorubicin through activation of p38 MAPK. Biochem Biophys Res Commun 406: 299–304. 21320468 10.1016/j.bbrc.2011.02.036

[30] Verma G, Datta M (2012) The critical role of JNK in the ER-mitochondrial crosstalk during apoptotic cell death. J Cell Physiol 227: 1791–1795. 21732347 10.1002/jcp.22903

[31] Yan MY, Chien SY, Kuo SJ, Chen DR, Su CC (2012) Tanshinone IIA inhibits BT-20 human breast cancer cell proliferation through increasing caspase 12, GADD153 and phospho-p38 protein expression. Int J Mol Med 29: 855–863. 22322382 10.3892/ijmm.2012.908

[32] Wu HL, Gao X, Jiang ZD, Duan ZT, Wang SK, et al (2013) Attenuated expression of the tight junction proteins is involved in clopidogrel-induced gastric injury through p38 MAPK activation. Toxicology 304: 41–48. 23220562 10.1016/j.tox.2012.11.020

[33] Zhang F, Ren G, Lu Y, Jin B, Wang J, et al (2009) Identification of TRAK1 (Trafficking protein, kinesin-binding 1) as MGb2-Ag: a novel cancer biomarker. Cancer Lett 274: 250–258. 18986759 10.1016/j.canlet.2008.09.031

[34] Bao WB, Ye L, Pan ZY, Zhu J, Du ZD, et al (2012) Microarray analysis of differential gene expression in sensitive and resistant pig to Escherichia coli F18. Anim Genet 43: 525–534. 22497274 10.1111/j.1365-2052.2011.02287.x

[35] Ohoka N, Yoshii S, Hattori T, Onozaki K, Hayashi H (2005) TRB3, a novel ER stress-inducible gene, is induced via ATF4-CHOP pathway and is involved in cell death. EMBO J 24: 1243–1255. 15775988 10.1038/sj.emboj.7600596 PMC556400

[36] Hayashi T, Saito A, Okuno S, Ferrand-Drake M, Dodd RL, et al (2005) Damage to the endoplasmic reticulum and activation of apoptotic machinery by oxidative stress in ischemic neurons. J Cereb Blood Flow Metab 25: 41–53. 15678111 10.1038/sj.jcbfm.9600005

[37] Oyadomari S, Mori M (2004) Roles of CHOP/GADD153 in endoplasmic reticulum stress. Cell Death Differ 11: 381–389. 14685163 10.1038/sj.cdd.4401373

[38] Marx J (2006) Cell biology. A stressful situation. Science 313: 1564–1566. 16973855 10.1126/science.313.5793.1564

[39] Zhao L, Ackerman SL (2006) Endoplasmic reticulum stress in health and disease. Curr Opin Cell Biol 18: 444–452. 16781856 10.1016/j.ceb.2006.06.005

[40] Puthalakath H, O’Reilly LA, Gunn P, Lee L, Kelly PN, et al (2007) ER stress triggers apoptosis by activating BH3-only protein Bim. Cell 129: 1337–1349. 17604722 10.1016/j.cell.2007.04.027

[41] Marciniak SJ, Yun CY, Oyadomari S, Novoa I, Zhang Y, et al (2004) CHOP induces death by promoting protein synthesis and oxidation in the stressed endoplasmic reticulum. Genes Dev 18: 3066–3077. 15601821 10.1101/gad.1250704 PMC535917

[42] Ord D, Meerits K, Ord T (2007) TRB3 protects cells against the growth inhibitory and cytotoxic effect of ATF4. Exp Cell Res 313: 3556–3567. 17707795 10.1016/j.yexcr.2007.07.017

[43] Zou CG, Cao XZ, Zhao YS, Gao SY, Li SD, et al (2009) The molecular mechanism of endoplasmic reticulum stress-induced apoptosis in PC-12 neuronal cells: the protective effect of insulin-like growth factor I. Endocrinology. 150: 277–285. 10.1210/en.2008-0794 18801901

[44] Du K, Herzig S, Kulkarni RN, Montminy M (2003) TRB3: a tribbles homolog that inhibits Akt/PKB activation by insulin in liver. Science 300: 1574–1577. 12791994 10.1126/science.1079817

[45] Jousse C, Deval C, Maurin AC, Parry L, Cherasse Y, et al (2007) TRB3 inhibits the transcriptional activation of stress-regulated genes by a negative feedback on the ATF4 pathway. J Biol Chem 282: 15851–15861. 17369260 10.1074/jbc.M611723200

[46] Seimon TA, Kim MJ, Blumenthal A, Koo J, Ehrt S, et al (2010) Induction of ER stress in macrophages of tuberculosis granulomas. PLoS One 5: e12772. 20856677 10.1371/journal.pone.0012772 PMC2939897

[47] Dogra N, Mukhopadhyay T (2012) Impairment of the ubiquitin-proteasome pathway by methyl N-(6-phenylsulfanyl-1H-benzimidazol-2-yl)carbamate leads to a potent cytotoxic effect in tumor cells: a novel antiproliferative agent with a potential therapeutic implication. J Biol Chem 287: 30625–30640. 22745125 10.1074/jbc.M111.324228 PMC3436308

[48] Xu L, Su L, Liu X (2012) PKCdelta regulates death receptor 5 expression induced by PS-341 through ATF4-ATF3/CHOP axis in human lung cancer cells. Mol Cancer Ther 11: 2174–2182. 22848091 10.1158/1535-7163.MCT-12-0602

[49] Chhabra R, Dubey R, Saini N (2011) Gene expression profiling indicate role of ER stress in miR-23a∼27a∼24–2 cluster induced apoptosis in HEK293T cells. RNA Biol 8: 648–664. 21593605 10.4161/rna.8.4.15583

[50] Kim I, Xu W, Reed JC (2008) Cell death and endoplasmic reticulum stress: disease relevance and therapeutic opportunities. Nat Rev Drug Discov 7: 1013–1030. 19043451 10.1038/nrd2755

[51] Mauro M, Zlatopolskiy A, Raife TJ, Laurence J (2004) Thienopyridine-linked thrombotic microangiopathy: association with endothelial cell apoptosis and activation of MAP kinase signalling cascades. Br J Haematol 124: 200–210. 14687031 10.1046/j.1365-2141.2003.04743.x

[52] Heitzmann D, Warth R (2008) Physiology and pathophysiology of potassium channels in gastrointestinal epithelia. Physiol Rev 88: 1119–1182. 18626068 10.1152/physrev.00020.2007

[53] Pabst MA, Wachter C, Holzer P (1996) Morphologic basis of the functional gastric acid barrier. Lab Invest 74: 78–85. 8569200

[54] Matsumoto M, Minami M, Takeda K, Sakao Y, Akira S (1996) Ectopic expression of CHOP (GADD153) induces apoptosis in M1 myeloblastic leukemia cells. FEBS Lett 395: 143–147. 8898082 10.1016/0014-5793(96)01016-2

[55] Wang XZ, Ron D (1996) Stress-induced phosphorylation and activation of the transcription factor CHOP (GADD153) by p38 MAP Kinase. Science 272: 1347–1349. 8650547 10.1126/science.272.5266.1347

[56] Wang K, Brems JJ, Gamelli RL, Holterman AX (2011) iNOS/NO signaling regulates apoptosis induced by glycochenodeoxycholate in hepatocytes. Cell Signal 23: 1677–1685. 21693187 10.1016/j.cellsig.2011.06.003

[57] Branger J, van den Blink B, Weijer S, Madwed J, Bos CL, et al (2002) Anti-inflammatory effects of a p38 mitogen-activated protein kinase inhibitor during human endotoxemia. J Immunol 168: 4070–4077. 11937566 10.4049/jimmunol.168.8.4070

[58] Nick JA, Avdi NJ, Young SK, Lehman LA, McDonald PP, et al (1999) Selective activation and functional significance of p38alpha mitogen-activated protein kinase in lipopolysaccharide-stimulated neutrophils. J Clin Invest 103: 851–858. 10079106 10.1172/JCI5257 PMC408145

[59] Maytin EV, Ubeda M, Lin JC, Habener JF (2001) Stress-inducible transcription factor CHOP/gadd153 induces apoptosis in mammalian cells via p38 kinase-dependent and -independent mechanisms. Exp Cell Res 267: 193–204. 11426938 10.1006/excr.2001.5248

[60] Fischer H, Koenig U, Eckhart L, Tschachler E (2002) Human caspase 12 has acquired deleterious mutations. Biochem Biophys Res Commun 293: 722–726. 12054529 10.1016/S0006-291X(02)00289-9

